# Influence of Anodizing Stages on the Preload Force of Implant–Abutment Screws and Their Benefits Regarding the Concept of Immediate Implant Placement—An In Vitro Study

**DOI:** 10.3390/ma15030776

**Published:** 2022-01-20

**Authors:** Florian Rathe, Paul Weigl, Jan Wasiak, Christoph Ratka, Holger Zipprich

**Affiliations:** 1 Department of Prosthodontics and Biomaterials, DANUBE Private University, 3500 Krems an der Donau, Austria; 2 Department of Prosthodontics, J.W. Goethe University, 60323 Frankfurt, Germany; weigl@em.uni-frankfurt.de (P.W.); janwasiak@gmx.net (J.W.); mail@zahnarzt-ratka.de (C.R.); 3 Independent Researcher, 64342 Seeheim-Jugenheim, Germany; holger.zipprich@gmail.com

**Keywords:** dental implant, abutment, preload force, abutment screw, anodization, immediate implant placement, screw loosening, abutment screw loosening

## Abstract

The tightening torque applied to a screw in a provisional restoration immediately after implant placement in a fresh extraction socket is often too low to gain sufficient preload force. Therefore, abutment screw loosening is a common complication. The aim of this study was to investigate whether it is possible to increase the preload force of a given tightening torque by anodizing parts of the implant–abutment complex. In test group 1 (TG1), only the abutment screw was anodized, in four different stages, whereas in test group 2 (TG2), the abutment and the threaded sleeve were anodized in four anodizing stages (TG2a–TG2d). The control group (CG) consisted of non-anodized components. The results were tested for normal distribution, and the components were subsequently parametrically analyzed using a linear model. Both test groups showed higher preload forces compared to the non-anodized control group. The CG obtained an average preload force of 390 N at a tightening torque of 35 Ncm. Comparable values were already obtained at a tightening torque of 20 to 30 Ncm in TG1c/D and TG2b/d. It can be concluded that anodization of abutment screws and components is an effective measure to increase the preload force of the abutment screws by a given tightening torque.

## 1. Introduction

Implant restorations in single tooth gaps are a well-documented treatment option, showing high long-term survival rates [1]. However, the need for complete bone healing before implant placement has been disproven [2,3,4,5]. This knowledge led to the concept of immediate implant placement into the fresh extraction socket with an immediate, nonfunctional, and provisional restoration. This concept has several advantages, such as a reduction in the overall treatment duration and the number of surgical interventions. Insertion torques of at least 20 Ncm were reported to be sufficient for immediate nonfunctional restoration [6]. Another systematic review revealed that most complications are of a technical nature, such as abutment screw loosening, with an incidence of up to 28% in one article [7]. Clinicians face a dilemma, especially in terms of immediately placed single implants with a low primary stability, since they might loosen the freshly placed implant if the tightening torque of the abutment screw is too high. However, if the applied torque too low, screw loosening will occur. The loosening of an abutment screw is likely to deteriorate into a complete fracture of the screw, and it might even cause biological complications via micro leakage at the implant–abutment interface [8,9,10]. A fracture of the abutment screw could even lead to the loss of the whole implant, with various causative factors being responsible. Tightening torque, which is applied by a torque wrench when mounting the abutment with the abutment screw, generates a preload force in the screw, clamping the abutment to the implant. Thus, in the case of the immediate restoration of immediately placed single implants, one should anticipate high preload forces with a low tightening torque to overcome the described obstacles. The preload force generated by the tightening torque depends on friction between the screwhead and the screwhead counterbore, as well as between the external threads of the abutment screw and the internal threads of the implant. The higher the friction, the lower the resulting preload force and vice versa. The relation between the friction coefficient and the preload force has been described by the equation T = KFd, with which the influence of friction on preload force can be computed. The preload F in a threaded fastener depends on the applied torque T, the nominal screw diameter d, and a constant “kappa” per an equation of the form (F = T/Kd). K depends on several factors, including the geometry of the screw thread itself and the coefficient of friction between the material of the screw and the material in which it is being installed. [11]. It has been shown that anodization, up to a certain thickness of the oxide layer, decreases the friction coefficient [12]. Anodization is an electrolytic oxidation process used to build a highly resistant oxide layer on the surface of passive metal parts. Passive metals such as aluminum, magnesium, titanium, zinc, and their alloys possess a natural oxide layer of approximately 0.05 nm. An additional oxide layer thickness of 38 to 167 nm after anodization is common [13]. Depending on the thickness of the oxide layer, titanium oxide appears in different colors due to its interference effect [13,14]. This effect of anodization is frequently used by manufacturers to color-code abutment screws, abutments, and implants.

The purpose of this study was to investigate whether it is possible to increase the preload force of a given tightening torque by anodizing, with a view to the clinical concept of immediate implant placement with immediate, nonfunctional restoration. Since different parts of the implant–abutment connection were anodized by the manufacturer for color-coding reasons, we analyzed whether anodization of the abutment screw only or all parts of the implant–abutment complex had an influence on the anticipated preload force compared to a non-anodized control implant–abutment complex. Furthermore, we aimed to analyze whether different anodization stages (blue, yellow, magenta, green) affected the anticipated preload forces compared to the non-anodized controls. We hypothesized that preload forces show no significant differences between the control group and the test groups.

## 2. Materials and Methods

**Figure 1:**
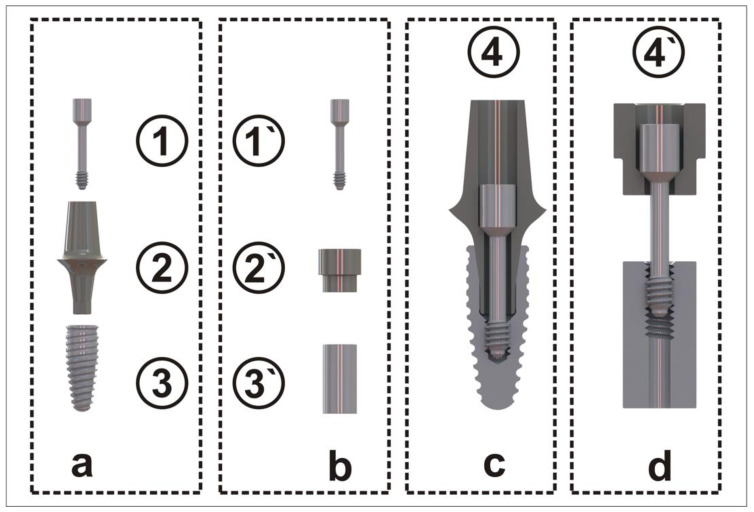
(**a**) Oral implant components and (**b**) analog test specimens (#1 and #1´ abutment screw; #2 abutment/#2´ abutment analog; #3 implant/#3´ thread sleeve); (**c**) composition of the components of an oral implant #4 and (**d**) composition of the components of the test specimens #4´.

**Figure 2:**
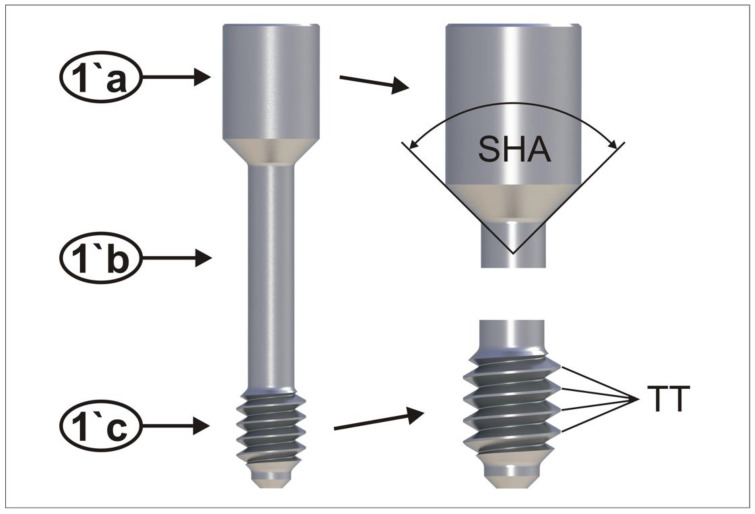
(**a–c**) parts of an abutment screw (1´a screwhead; 1´b screw shaft; 1´c screw thread) screwhead angle (SHA) of 90 degrees; 4 thread turns (TT´s).

Test specimens including an abutment screw, an abutment analog, and a thread sleeve resembling the implant (Figure 1) were custom manufactured. All parts were turned with the same lathe (EmcoTurn 120, EmcoTronic TM02; EMCO Maier GmbH, Pleidelsheim, Germany). The test screws had a screwhead angle (SHA) of 90 degrees, 4 thread turns (TTs, Figure 2), a thread pitch of 0.35 mm, a metric M1.6 external thread (ISO thread DIN 13), and were manufactured using Grade 5 titanium. The abutment analog was made of Grade 5 titanium harboring the screwhead counterbore in its center. The screwhead counterbores were turned at angles corresponding to the screwhead angle of the abutment screws. The abutment screw configurations were based on commercially available abutment screws. In total, 90 abutment screws were analyzed in this study.

After manufacturing, the test specimens were cleaned to remove swarf, remnants of cooling liquid, and other contaminants. They were subsequently degreased using an ethanol wash (70%), cleaned in an ultrasonic bath, and steam blasted.

Each specimen was inspected after cleaning. Screwhead angles were controlled on a coordinate measuring table (060-366.006—Ernst Leitz GmbH, Wetzlar, Germany) driven by two digital micrometer heads (350-273-10—Mitutoyo Kawasaki, Sakado, Japan). Screwhead counterbores were checked with the same method after a central cut through their axes. The accepted fabrication tolerance was +0.5 degrees for screwhead angles and −0.5 degrees for screwhead counterbores. The internal threads of the threaded sleeves were manufactured with tolerance class 6H (DIN 965-1), and the external threads of the abutment screws were manufactured with tolerance class 6g (DIN 965-1).

**Figure 3:**
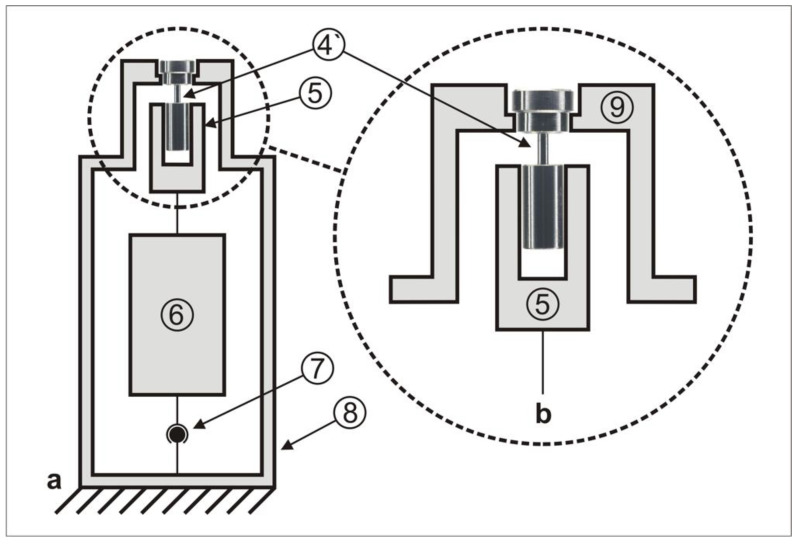
Measurement arrangement (**a**) with enlargement (**b**) (#4’: “simulated implant–abutment connection”; #5: collet chuck for thread sleeve; #6: combined force and torque sensor; #7: cardan joint; #8: housing of measurement station; #9: fixture of abutment analog).

The measurement arrangement is shown in Figure 3, and it was first described elsewhere [15]. When the abutment screw was screwed into the thread sleeve and the screwhead’s counterbore engaged in the abutment analog, the collet chuck for the thread sleeve (Figure 3, #5) was pulled toward the fixture of the abutment analog (Figure 3, #9).

In addition to the simulation suitability of the implant components (Figure 1, #1`, #2`, #3`), a correct geometric positioning of the components had to be ensured by the measurement arrangement. Therefore, a cardan joint (Figure 3, #7) was placed between the sensor (Figure 3, #6) and the measurement station (Figure 3, #8). The cardan joint leveled out possible axial deviations between the components due to shearing forces, which could have led to an undesirable increase in friction, thus influencing the preload force of the screw [15].

To avoid this, components #5 and #9 approached each other; they were joint-friction locked by means of the measurement station housing (Figure 3, #8), the cardan joint (Figure 3, #7), and the combined force and torque sensor (Figure 3, #6) (M-2396, 2 Nm/500 N as a one-off production; Lorenz Messtechnik GmbH, Alfdorf, Germany).

Five torque wrenches (Biodenta Swiss AG, Berneck, Switzerland) were used in this experiment. The torque was manually adaptable by turning the handle. Each torque wrench was calibrated to one of the following tightening torques: M_1_ = 15 Ncm, M_2_ = 20 Ncm, M_3_ = 25 Ncm, M_4_ = 30 Ncm, or M_5_ = 35 Ncm. The electronic calibration of the torque wrenches was performed by a static torque sensor (D-2452, 1 Nm, Lorenz Messtechnik GmbH).

After mounting the threaded sleeve and the abutment analog onto their attachments, the two devices were vertically aligned using spherical head screws. Subsequently, the abutment screw was inserted into the screwhead counterbore and turned 4 times in line with the number of its threads. The objective of this step was to ensure that all thread turns touched the internal threads before the screws were tightened. After zero-value calibration, the screw connection was put under a preload of 40–60 N to prevent the screw from penetrating too deeply into the thread sleeve. If the screw penetrates to deeply, there is a risk that its thread will outrun the thread. This preload was set by turning the threaded fixture of the abutment analog (Figure 3, #9) counterclockwise (Figure 3, #8). The screw was tightened in 5 steps (M_1_ to M_5_) using the torque wrench. Prior to each step, the respective torque wrench was calibrated.

Differently anodized (blue, yellow, magenta, green) abutment screws, thread sleeves, and abutment analogs were tested in different combinations (Table 1). The thickness of the oxide layer increased in line with the anodization stages from blue to green (blue = 50.5 nm, yellow = 102.6 nm, magenta = 121 nm, and green = 167 nm) [13]. Only abutment screws were anodized in test group 1 (TG1), with the following subgroups depending on the anodization stage: TG1a = blue, TG1b = yellow, TG1c = magenta, and TG1d = green, whereas all components (abutment screw, thread sleeve, and abutment analog) were anodized in test group 2 (TG2) with the subgroups TG2a = blue, TG2b = yellow, TG2c = magenta, and TG2d = green. Each of the 9 groups was tested with a subset of 10 screws. All test specimens were compared with the non-anodized control group (CG) and anodized elsewhere (ETG- Elektronik GmbH, Weiterstadt, Germany).

In brief, the anodization of the components was carried out under room temperature with an electrolyte (titanium tinting electrolyte; article number: 3030400102; Wieland-Edelmetalle, Pforzheim, Germany) in a beaker (250 mL). The components were electronically contacted with a titanium wire at a surface not important for the experiment and individually immersed in the electrolyte. To avoid air bubbles, which could hinder anodization, the inner parts of the thread sleeve and the abutment analog were filled with the electrolyte by a syringe. A titanium sheet (Grade 2) served as cathode and the components were loaded with a specific voltage (blue = 28 V; yellow = 63 V; magenta = 78 V; green = 102 V) via a power source (2× PeakTech DC Dual Power supply 6060, serially connected; PeakTech, Ahrensburg, Germany) over a period of 60 s. After the anodization process, components were purged with distilled water and isopropanol (70%).

The statistical evaluation was supported by the Department of Biostatistics of the Goethe University Frankfurt a.M., Germany. The measurement data (e.g., preload force [N]) of the test groups TG1a–d and TG2a–d were each tested using the Kolmogorov–Smirnov–Lilliefors test (R function lillie.test). To use a parametric evaluation, the data must be normally distributed. To assess the normal distribution, the R package nortest (nortest_1.0-4) was used in the R program. The data were parametrically determined by a linear model with the R package PMCMR (PMCMR_4.1) and evaluated. The linear model is based on the linear regression method.

## 3. Results

**Figure 4:**
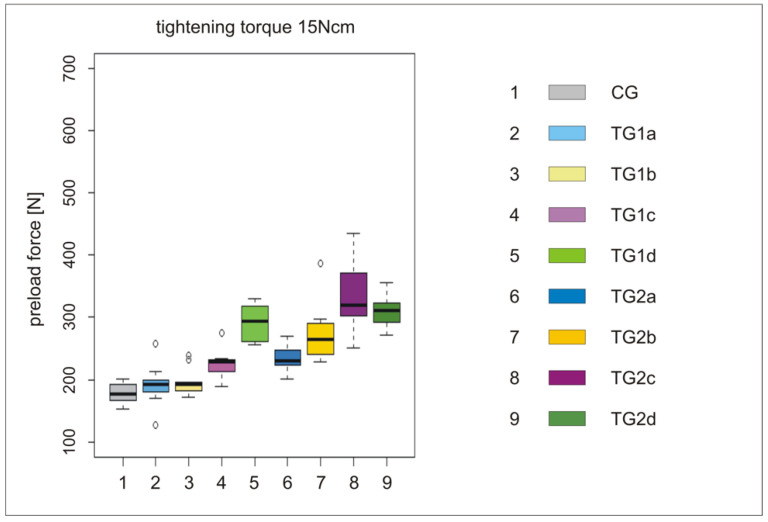
Boxplots of groups CG, TG1a–d, and TG2a–d with preload forces at a torque force of 15 Ncm, (**left**); a detailed legend of the x-axis (**right**).

**Figure 5:**
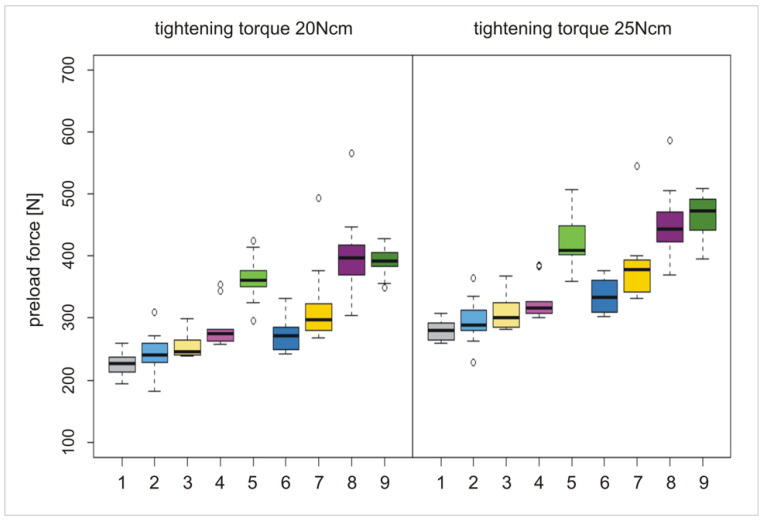
Boxplots of groups CG, TG1a–d, and TG2a–d with preload forces at a torque force of 20 Ncm, (**left**); boxplots of groups CG, TG1a–d, and TG2a–d with preload forces at a torque force of 25 Ncm (**right**).

**Figure 6:**
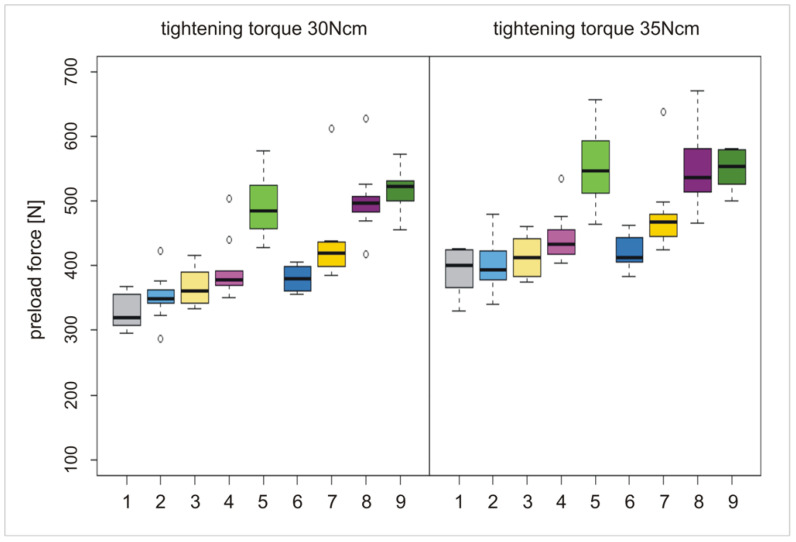
Boxplots of groups CG, TG1a–d, and TG2a–d with preload forces at a torque force of 30 Ncm, (**left**); boxplots of groups CG, TG1a–d, and TG2a–d with preload forces at a torque force of 35 Ncm (**right**).

All the results of the different tightening torques (M_1_ = 15 Ncm to M_5_ = 35 Ncm) are summarized in Table 2. Test groups (TG1a–d and TG2 a–d) were compared with the control group (CG, Figure 4, Figure 5 and Figure 6). Preload forces of TG1c, TG1d, and TG2b–d were statistically significantly higher compared to CG (Table 2). However, the highest preload forces were measured for the anodization stages TG1d, TG2c, and TG2d compared to CG, independently of the applied torque force. Control group CG exhibited an average preload force of 390 N if a tightening torque of 35 Ncm was used. A similar preload force was achieved at TG1d with a torque of 25 Ncm, whereas at TG2c and TG2d, a similar preload force was realized with a torque of only 20 Ncm (Table 2, Figure 5). The preload forces of TG1d, TG2c, and TG2d with a tightening torque of 35 Ncm were, on average, 150 N higher than the preload force of CG (Table 2, Figure 6).

In particular, the anodization stages green (TG1d and TG2d) and magenta (TG1c and TG2c) showed a high increase in preload forces of the abutment screws. Barring the fact that the preload (556.08 N) in TG1d was slightly greater than that inTG2d (550.81), the preload of all groups in which only abutment screws (TG1a–c) were anodized was lower than that in which all components were anodized (TG2a–c).

Additionally, it was observed that preload forces increased with the applied tightening torque, irrespective of the degree of anodization (data not shown). This phenomenon had already been found in previous studies [15,16].

## 4. Discussion

The preload forces of TG1c–d and TG2a–d were significantly higher compared to those of CG. Therefore, our working hypothesis must be rejected. The purpose of this study was to investigate whether it is possible to increase the preload force of a given tightening torque by anodizing with a view of the clinical concept of immediate implant placement with immediate, nonfunctional restoration. Particularly, the anodization stages green (TG1d and TG2d) and magenta (TG1c and TG2c) showed high potential to increase the preload forces of the abutment screws. All groups with an anodization of the abutment screw only (TG1a–d) showed lower preload forces compared to the groups with anodization of all components (TG2a–d). Thus, TG2c and TG2d screws showed the highest preload forces. However, group TG1d revealed preload forces similar to TG2c and TG2d, although only the abutment screw was anodized, while TG1c appeared to be significantly less effective at increasing the preload force. This phenomenon might be explained by the thickness of the titanium oxide layer. Since its thickness increases in line with the anodization stages from blue to green, it seems that this layer is destroyed by wear to a certain extent, at least when the screw is tightened. The anodization layer of TG1d does not seem to be destroyed completely after a screw is tightened once, thus resulting in a higher preload force compared to the TG1c group. However, at a certain thickness of the oxide layer, this effect seems to be negligible, since differences in preload forces between TG2c and TG2d are only minor. This is in accordance with a study investigating the effect of anodization on the friction coefficient, which established that, at approximately 100 nm thickness, the friction coefficient increased again [12].

As described above, the tightening torque of the provisional restorations on immediately placed implants should neither be too high nor too low in order not to jeopardize the primary stability of the implant or promote abutment screw loosening. A minimum torque of 15–20 Ncm has been suggested to limit screw loosening, but this has never been proven to be sufficient [7]. However, a tightening torque of 15 Ncm applied at an immediately placed implant with an insertion torque of 20 Ncm might be too low to avoid screw loosening, especially if the manufacturer recommends a tightening torque of 20 Ncm or even more to achieve a sufficient preload force. For these instances, this study provides evidence for manufacturers suggesting that the use of an anodized abutment screw is sufficient to anticipate an equal preload force with less torque, as well as evidence that all components of the implant–abutment should be anodized. Patients’ quality of life would improve if implant-fixed provisionals were provided during the implant healing period through the measure of anodization. The alternative, a removable provisional, often covers big parts of the palate, which could provoke phonetic problems as well as gustatory restrictions.

The conclusions of the present study are limited to the tested screw configurations (e.g., screwhead angle and thread pitch), and they are valid for a single tightening procedure with a dry implant lumen only. However, it can be assumed that other screw configurations might respond in a similar fashion.

Since clinical settings require the tightening and untightening of a screw-retained crown at least twice (nonfunctional restoration and after the healing period the definitive, functional restoration), further research should focus on the effect of anodization on the preload force after repeated tightening of the abutment screw. This would provide added value for clinicians in deciding whether to use a new abutment screw to fix the definitive restoration or to continue with the one already in use for the provisional nonfunctional crown.

## 5. Conclusions

Within the limits of the study, it can be concluded that the anodization of abutment screws and components is an effective measure to increase the preload force of the abutment screws by a given tightening torque. Anodization stages magenta and green specifically anticipated higher preload forces compared to our non-anodized controls. Furthermore, anodization of all components seems more effective in enhancing the preload force compared to the anodization of the abutment screw only.

## Figures and Tables

**Table 1:** Anodization and anodization stage of the components.

Surface Finishing	Groups			
Anodization of none of the components	CG			
	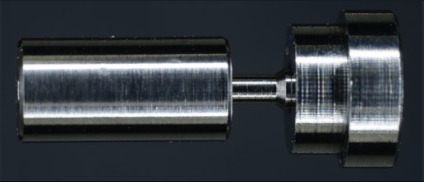			
Anodization of the abutment screw only	TG1a	TG1b	TG1c	TG1d
	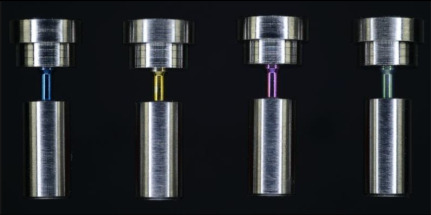			
Anodization of all components	TG2a	TG2b	TG2c	TG2d
	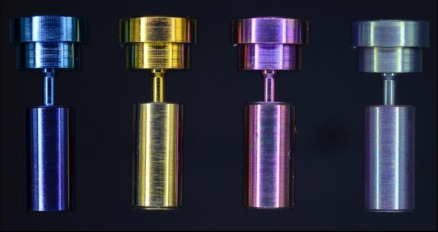			

**Table 2:** Average preload forces based on group and tightening torque. *p*-values refer to the comparison with the control croup CG.

Average					
Groups	15 Ncm	20 Ncm	25 Ncm	30 Ncm	35 Ncm
CG	178.22	226.94	281.02	327.81	390.83
TG1a *p*-value	191.93 0.341	243.49 0.373	294.88 0.453	350.90 0.219	400.57 0.617
TG1b *p*-value	196.18 0.213	256.95 0.108	311.64 0.099	368.56 0.031	416.06 0.196
TG1c *p*-value	226.35 0.001	285.13 0.002	327.49 0.013	395.03 <0.001	444.45 0.007
TG1d *p*-value	294.39 <0.001	363.39 <0.001	426.81 <0.001	493.76 <0.001	556.08 <0.001
TG2a *p*-value	233.40 <0.001	271.80 0.017	335.09 0.004	380.78 0.006	421.85 0.114
TG2b *p*-value	274.92 <0.001	321.80 <0.001	385.39 <0.001	434.59 <0.001	480.07 <0.001
TG2c *p*-value	329.65 <0.001	405.43 <0.001	455.76 <0.001	502.34 <0.001	549.39 <0.001
TG2d *p*-value	309.48 <0.001	392.15 <0.001	462.41 <0.001	515.31 <0.001	550.81 <0.001

## Data Availability

Further data available upon request: weigl@em.uni-frankfurt.de.
